# Improved identification of concordant and discordant gene expression signatures using an updated rank-rank hypergeometric overlap approach

**DOI:** 10.1038/s41598-018-27903-2

**Published:** 2018-06-25

**Authors:** Kelly M. Cahill, Zhiguang Huo, George C. Tseng, Ryan W. Logan, Marianne L. Seney

**Affiliations:** 1 Department of Biostatistics, Graduate School of Public Health, University of Pittsburgh, Pittsburgh, PA USA; 2 Department of Biostatistics, College of Public Health & Health Professions College of Medicine, University of Florida, Gainsville, FL USA; 3 Department of Computational and Systems Biology, School of Medicine, University of Pittsburgh, Pittsburgh, PA USA; 4 Department of Psychiatry, School of Medicine, University of Pittsburgh, Pittsburgh, PA USA; 5 Translational Neuroscience Program, School of Medicine, University of Pittsburgh, Pittsburgh, PA USA; 6 The Center For Systems Neurogenetics of Addiction, The Jackson Laboratory, Bar Harbor, ME USA

## Abstract

Recent advances in large-scale gene expression profiling necessitate concurrent development of biostatistical approaches to reveal meaningful biological relationships. Most analyses rely on significance thresholds for identifying differentially expressed genes. We use an approach to compare gene expression datasets using ‘threshold-free’ comparisons. Significance cut-offs to identify genes shared between datasets may be too stringent and may miss concordant patterns of gene expression with potential biological relevance. A threshold-free approach gaining popularity in several research areas, including neuroscience, is Rank–Rank Hypergeometric Overlap (RRHO). Genes are ranked by their *p*-value and effect size direction, and ranked lists are compared to identify significantly overlapping genes across a continuous significance gradient rather than at a single arbitrary cut-off. We have updated the previous RRHO analysis by accurately detecting overlap of genes changed in the same and opposite directions between two datasets. Here, we use simulated and real data to show the drawbacks of the previous algorithm as well as the utility of our new algorithm. For example, we show the power of detecting discordant transcriptional patterns in the postmortem brain of subjects with psychiatric disorders. The new R package, RRHO2, offers a new, more intuitive visualization of concordant and discordant gene overlap.

## Introduction

Comparing patterns of gene expression between experimental groups is often useful for exploring common molecular pathways potentially involved in specific biological processes. Current approaches typically use correlations or enrichment between multiple sets of genes employing statistical cutoffs (e.g., *p* < 0.05 or 5% false discovery rate) to derive a list of differentially expressed genes. In large-scale gene expression experiments (microarrays, RNA-seq, etc), gene sets are narrowed from thousands to hundreds or maybe tens of genes, which represent the most significant genes with presumably the highest relevance. These gene sets are then characterized based on pathways, functions, or structural characteristics from popular annotated databases. For enrichment, statistical significance is typically determined by the hyper-geometric distribution, or one-sided Fisher’s exact test. While these are intuitive and readily implemented, genome-wide sequencing experiments generate a continuous range of expression differences of thousands of genes, such that ‘hard’ arbitrary cutoffs could impact the veracity of significance (e.g., conservative vs. lenient), consequently discarding subtle changes and relevant biological information.

Often, researchers aim to compare two separate differential gene expression datasets to identify overlap trends. For instance, one might examine differential gene expression between stressed and non-stressed mice in two different brain regions. In this example, there are two separate lists of differentially expressed genes (one for each brain region). To identify genes that are differentially expressed in both brain regions, genes are identified using a strict significance cutoff in each dataset (e.g., *p* < 0.05 in both); lists of genes are then compared to reveal overlapping pathways between groups. While these approaches have proved valuable, they are greatly impacted by experimental design, sample size, and perturbation, and may miss relevant biological information.

A complementary approach to using significance cutoffs when comparing two differential expression datasets is a threshold free algorithm called rank-rank hyper-geometric overlap (RRHO). RRHO enables the direct comparison of separate gene sets to reveal overlapping trends. For each dataset, RRHO ranks the entire gene list by differential expression *p*-values and effect size direction. For each dataset, these ranks are arranged vertically or horizontally to graphically display the significance of the overlap intensity. Thus, the algorithm steps through each ranked gene to obtain significance values representing the number of overlapping genes and the resulting significance matrix is visualized using a heatmap. Each point represents the significance of the number of overlapping genes via the hyper-geometric distribution^1^. The final heatmap of the RRHO represents potentially overlapping biologically relevant signatures. RRHO can be used to compare differential gene expression profiles between multiple species, different microarray platforms, model systems, and experimental perturbations, such as genetic manipulations or drug treatments. RRHO provides an intuitive and comprehensive tool for identifying common patterns of gene expression between multiple datasets combined with graphical representation which summarizes the intensity of these patterns. The software for conducting RRHO is publically available as a Bioconductor R package and other web-based applications.

Applications of RRHO have been widely used to determine gene enrichment between multiple datasets where the intention is to reveal common molecular signatures of independent groups^2–6^. The input for RRHO requires gene significance values derived from group comparisons and including the effect size direction is valuable for the assessment and interpretation of the overlaps. Currently, there are two representations for RRHO: Enrichment and Two-sided. For Enrichment, each pixel in the heatmap represents higher than expected overlap between gene sets and the overlaps are represented in quadrants, where the bottom left and top right represent up-regulated and down-regulated genes in common between both gene sets, respectively (i.e., concordance). This is the option most frequently applied, and most studies implementing this approach are designed to discover concordance. The representation of the top left and bottom right quadrants was intended to identify overlapping genes with opposite expression patterns (i.e., discordance). The algorithm originally proposed by Plaiser and colleagues^1^ suggests ‘hotspots’ occurring within these other quadrants adequately denotes genes up-regulated in the one dataset and down-regulated in the other. However, the interpretation of these quadrants has been more challenging. Similarly, the Two-sided approach leads to ambiguous results due to the potential inverse of positive and negative heatmap values. Values in the top left and bottom right quadrants must be interpreted with caution.

We propose a modified algorithm to improve the efficiency, representation, and interpretation of these powerful RRHO approaches. First, we demonstrate the limitations of the current RRHO approaches to reveal discordant gene signatures. Second, we update the RRHO algorithms to enhance the Enrichment and Two-sided approaches in order to provide more intuitive representations, which should lead to improved implementation of these applications. Third, we propose an additional approach to the RRHO package which uses the Fisher exact test effect size to rank the genes rather than *p*-values. We compared the performance of our Stratified representation with the Enrichment and Two-sided algorithms using simulated and experimental datasets. Lastly, we made the RRHO2 package publicly available on Bioconductor.

## Methods

### Original RRHO implementation

In order to compare the concurrence of the top differential expression genes from two independent studies, S1 and S2, the RRHO algorithm utilizes the hypergeometric distribution to determine the degree of overlap between particular subsets of genes. The hypergeometric distribution is as follows:

1 $$h(k;s,M,N)=\,\frac{(\begin{array}{c}M\\ k\end{array})(\begin{array}{c}N-M\\ s-k\end{array})}{(\begin{array}{c}N\\ s\end{array})}$$

The distribution describes the number of successes in a sequence of $s$ draws from a finite population $N$ without replacement. $k$ is the number of successes in $s$ draws and *M* is the number of success in the whole population. The expected number of successes from the hypergeometric distribution is:

2 $$E(k)=\,\bar{k}=s\frac{M}{N}$$

**Figure 1:**
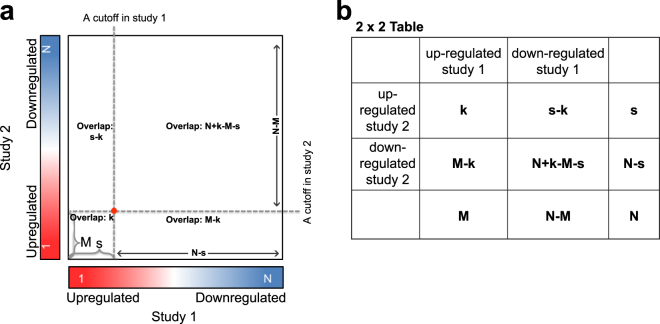
Representation of RRHO. (**a**) For study 1 on the x-axis, all genes are ranked based on their *p*-values and effect size direction; the most significantly up-regulated genes are the farthest to the left on the x-axis (red), the most significantly down-regulated genes are the farthest to the right on the x-axis (blue), and the relatively unchanged genes are in the middle of the x-axis (white). Similarly, the genes in study 2 are ranked from most significantly up-regulated (bottom, red), most significantly down-regulated (top, blue), unchanged (middle, white) in between. (**b**) A 2 × 2 contingency table. Using the table, calculation of the hypergeometric *p*-value can be computed directly.

For the original RRHO application, a total of N common genes from two studies are ranked by their degree of differential expression (DDE) (i.e., −log_10_(*p*-value) × effect size direction), respectively in each study. Under this definition, up-regulated genes have positive DDE and down-regulated genes have negative DDE. For study 1, the genes are ranked according to their DDE along the x-axis, where the far left gene has largest DDE (up-regulated, red color) and the far right gene has the smallest DDE (down-regulated, blue color) (Fig. 1). The changing point of the DDE direction (same as the effect size direction) lies around the middle of x-axis (white color). Study 2 is similarly ranked on the y-axis with the genes with the largest DDE at the far bottom and smallest one at the far top (Fig. 1). Therefore, a hot spot (red pixel in Fig. 1) towards the center of the plot indicates overlap in genes that are less significantly DE, while a pixel towards the outer corners indicates overlap among genes that are more significantly DE in each study.

Arbitrary DDE cutoffs for study 1 and study 2 are represented as the red dot in Fig. 1, and the actual cutoff value can be obtained by projecting the red dot onto the x and y axes for studies 1 and 2, respectively. By applying these gene selection cutoffs and counting from largest DDE in study 1 and 2, a list of $s$ genes from study 1 and a list of $M$ genes from study 2 can be obtained. Denote $k$ as the number of overlapping genes between $s$ genes from study 1 and $M$ genes from study 2. To formally assess the significance of the overlap between $s$ genes from study 1 and $M$ genes from study 2, a hypergeometric *p*-value can be obtained by using the probability that the number of overlapped genes is as extreme or more extreme than the observed overlapped genes, $k$:

3 $$\,H(k;s,M,N)=\sum _{j=k}^{s}h(j;s,M,N)$$

For each possible combination of DDE cutoffs in study 1 and study 2 (each dot in the main rectangle region in Fig. 1**)**, we can obtain a hypergeometric *p*-value. The resulting *p*-values are −log_10_ transformed and visualized using the same x-axis and y-axis in Fig. 1 as coordinates. This is known as the RRHO Enrichment method^1^. The hypergeometric distribution is symmetric:

4 $$H(k;s,M,N)=H(N+k-M-s;N-s,N-M,N)$$

**Figure 2:**
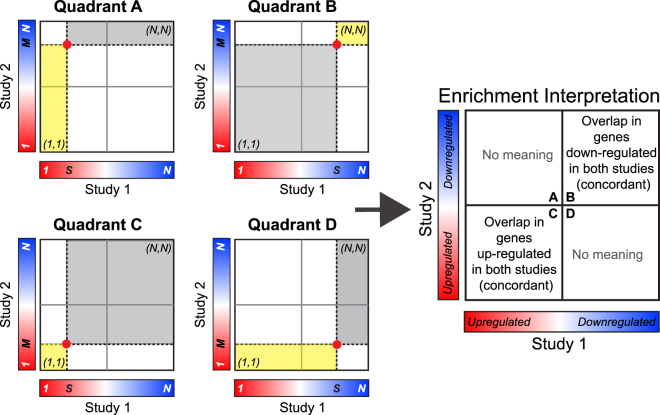
Interpretation of RRHO Enrichment method. A significant heatmap signal in either quadrants B or C is intuitive, representing overlap in genes changed in the same direction in the two studies. However, interpretation of signal in quadrants A or D is not intuitive. For instance, a signal in quadrant A indicates overlap in genes up-regulated in study 1 with genes that are down-regulated, not changed, and up-regulated in study 2. Similarly, a signal in quadrant D indicates overlap in genes up-regulated in study 2 with genes that are down-regulated, not changed, and up-regulated in study 1. Thus, using the enrichment method, signal in quadrant A or quadrant D is not intuitive.

Thus, one would expect the identical hypergeometric *p*-value by applying the same DDE cutoffs and by counting from smallest DDE in both study 1 and 2. Therefore, the Enrichment method gives great detail into the overlap among genes that share effect size direction. The resulting RRHO heatmap can be interpreted as in Fig. 2. A significant hypergeometric *p*-value for overlap in quadrant B of Fig. 2 is interesting, as it represents a significant concurrence of down-regulated genes in both study 1 and study 2. Similarly, a significant hypergeometric *p*-value for overlap in quadrants C of Fig. 2 is interesting, as it represents a significant concurrence of up-regulated genes in both study 1 and study 2. However, the RRHO Enrichment method leaves the overlap that is in quadrants A and D of Fig. 2 uninteresting because it lacks the symmetry property. For example, one can never interpret a significant hypergeometric *p*-value in quadrant A of Fig. 2 as a significant concurrence of up-regulated genes in study 1 and down-regulated genes in study 2. This is one limitation for RRHO application.

### Improved RRHO implementation – “Stratified method”

**Figure 3:**
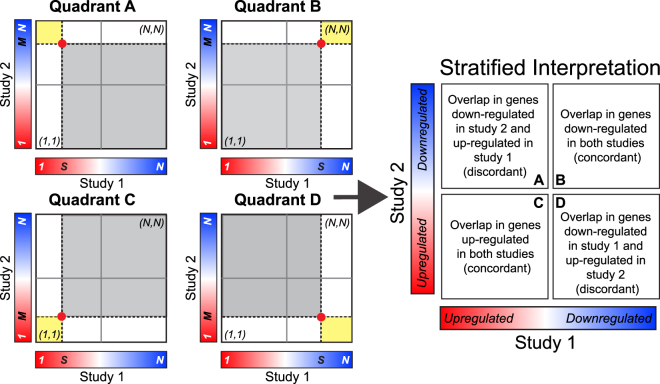
Interpretation of the Stratified RRHO method. A hotspot in quadrant A indicates overlap in genes up-regulated in study 1 and down-regulated in study 2. A hotspot in quadrant B indicates overlap in genes down-regulated in both studies. A hotspot in quadrant C indicates overlap in genes up-regulated in both studies. A hotspot in quadrant D indicates overlap in genes down-regulated in study 1 and up-regulated in study 2.

Previous applications of RRHO have been designed to quantify the significance of overlap by gene lists between two datasets that are both up-regulated or both down-regulated. For these experimental designs, the Enrichment method is appropriate. Recently, we became interested in gene lists with opposite effect size directions between studies (Quadrant A or Quadrant D in Fig. 2). While a simple examination of effect size directions indicated that many of the same genes were changed in opposite directions in our datasets, the Enrichment RRHO method never returned a hotspot in Quadrants A or D. A close examination of the RRHO Enrichment algorithm revealed that it is unable to detect meaningful signal in Quadrants A and D (see description above). The Two-sided RRHO method (see Supplementary Figures 1 and 2) is problematic as well, as only a negative overlapping score in Quadrant A or Quadrant D provides an over-enrichment interpretation. This motivated us to provide an update to the original algorithm such that these discordant quadrants are meaningful and to prevent further misinterpretation of RRHO results. We call our updated algorithm the “Stratified method”, in which degree of overlap is based on quadrant specific analyses. Specifically, instead of always counting from largest DDE to the cutoff points for all quadrants, we designed a new scheme such that, for each quadrant, we count from the outward corner to the cutoff point to define $s$ genes from study 1 and $M$ genes from study 2. Under this design, we always use the following formula to calculate the *p*-value for over-enrichment, but all four quadrants are biologically interesting (see Fig. 3 for detailed interpretations):

5 $$H(k;s,M,N)=\sum _{j=k}^{s}h(j;s,M,N)$$

To further distinguish these four quadrants, we place a white strip at the DDE changing point from up-regulation to down-regulation, as shown in the right plot of Fig. 3. The Stratified method clearly indicates where the overlap between the two DE lists occurs, diminishing any possibility of reporting a false positive or false negative  result of overlap in the discordant directions. The new scheme is implemented in an R package called RRHO2, which is publicly available at Bioconductor. As in the original Enrichment method, the user can select a scale in −log_10_(*p*-value) or −log_e_(*p*-value). The scale max using log base 10 will be approximately half the scale max using natural log; however, the significance of the result does not change based on the selected scale.

Using signed −log_10_(*p*-value) from the hypergeometric distribution sometimes suffers from the problem that all pixels for the heatmap may be “significant”. As sample size increases, it becomes easier to detect significance using *p*-values, creating potential for spurious finding. To overcome this problem, the new RRHO2 package also includes an option for calculating the significance of overlap according to the effect size (log odds ratio) from the Enrichment 2 × 2 table (see Fig. 1b), rather than by *p*-values. We call this the Log Odds Ratio method. The use of effect sizes is independent of sample size, so the risk of seeing statistical significance only because of large sample size, despite minimal biological difference, is diminished. In the original RRHO implementations, each pixel on the heatmap represents a −log_10_(*p*-value) from the one-sided test of $k > \bar{k}$. This same idea can be extended to use the log odds ratio obtained in fisher’s exact test. Each pixel now represents the log odds ratio at a specific threshold,$\,s$ and $M$. As seen in Fig. 1b, the red dot represents one pixel location. Each pixel location will split the heatmap into four quadrants as in Fig. 1b. The log odds ratio can be calculated directly as:

6 $$\begin{array}{c}\theta =\,\mathrm{log}\{\frac{k(N+k-M-s)}{(s-k)(M-k)}\}\,for\,quadrants\,B\,and\,C\\ \theta =\,\mathrm{log}\{\frac{(s-k)(M-k)}{k(N+k-M-s)}\}\,for\,quadrants\,A\,and\,D\end{array}$$

The odds ratio is the ratio of the odds of overlap in the concordant quadrants compared to the odds of overlap in the discordant quadrants; the numerator and denominator are switched when examining odds ratio in concordant or discordant quadrants. We count from the outward corner of the cut-off point to define *s* genes from study 1 and *M* genes from study 2 (see the 2 × 2 table in Fig. 1b). We have two formulas for the odds ratio to keep the interpretation of a positive effect size (indicating significant overlap) consistent among all the quadrants. Each pixel location will return a different odds ratio, and the heatmap will show the intensity of the effect size at each pixel. One limitation with the Log Odds Ratio representation of RRHO is that the bounds on the log odds ratio extend from negative infinity to infinity. To overcome this limitation, we bound the log odds ratio by the minimum and maximum finite values, causing the heat map to appear less continuous than the *p*-value heat maps.

### Simulation

To demonstrate the Stratified method, we randomly generate differential expression *p*-values for a simulated list of 10000 genes for experiment X and experiment Y. The steps are as follows:

1. Let $S=2$ be the number of studies representing experiment X and experiment Y. $G$ = 10, 000 is the total number of genes, and $N$ = 20 be the number of cases and controls ($2N$ samples in total).
2. We simulate correlated gene structures, and first assume no effect size for all genes in the two studies. We sample expression levels with genes following the procedure in^7^.
  - Sample 200 gene clusters with 20 genes in each cluster and the remaining 6,000 genes are uncorrelated. Denote by ${C}_{g}$ the cluster membership indicator for gene $g$, e.g. ${C}_{g}$ = 1 indicates gene g is in cluster 1, whereas ${C}_{g}$ = 0 indicates gene $g$ is not in any gene cluster.
  - For cluster *c* and study *s*, sample ${A^{\prime} }_{cs}\, \sim \,{W}^{-1}$(Ψ, 60), where 1 ≤ c ≤ 200, Ψ = $0.5{I}_{20x20}+0.5{J}_{20x20}$,$\,{W}^{-1}$ denotes the inverse Wishart distribution, I is the identity matrix and J is the matrix with all elements equal to 1. Then ${A}_{cs}\,\,$is calculated by standardizing ${A^{\prime} }_{cs}\,\,$such that the diagonal elements are all 1′s. The covariance matrix for gene cluster $c\,\,$in study $s$ is calculated as $\sum _{cs}=\,{\sigma }^{2}{A}_{cs}$, where σ is a tuning parameter we vary in the evaluation.
  - Denote the indices of the 20 genes in cluster $c$ as ${g}_{c1},\ldots ,\,{g}_{c20}$ (i.e. ${C}_{{g}_{cj}}=c$ where $1\le c\le C(C=200),$ and $1\le j\le 20.\,\,$Sample expression levels of genes in cluster $c$ from sample $n$ in study $s$ as $({X\text{'}}_{{g}_{c1}},\ldots ,{X\text{'}}_{{g}_{cn}})\, \sim \,MVN(0,\,\sum _{cs}),$ where $1\le n\le 2N$ and $1\le s\le S.$
  For any uncorrelated gene $g$ with ${C}_{g}=0,$ sample the expression level for sample $n$ in study $s$ as ${X^{\prime} }_{gsn}\, \sim \,N(0,\,{\sigma }^{2}),\,\,$where $1\le n\le 2N$ and $1\le s\le S.$
3. Sample DE genes, effect sizes, and their differential expression directions.
  - For any DE gene $g\,(1\le g\le {G}_{1}$), sample gene level effect size ${\theta }_{g} \sim {N}_{0.5+}(1,1),$ where ${N}_{a+}\,\,$denotes the truncated Guassian distribution within interval ($a,\infty ).$
  - Sample ${d}_{g}\, \sim \,Ber(0.5),\,\,$where $1\le g\le {G}_{1}.$ Here ${d}_{g}\,$controls effect size direction for gene $g$.
4. Add the effect sizes to the gene expression levels sampled in step 2c. For controls $(1\le n\le N),\,\,$set the expression levels as ${X}_{gsn}={X^{\prime} }_{gsn}$. For cases $(N+1\le n\le 2N),\,\,$if $1\le g\le {G}_{1}$, set the expression levels as ${X}_{gsn}={X^{\prime} }_{gsn}$ + ${(-1)}^{{d}_{g}}{\theta }_{gs.}$

**Figure 4:**
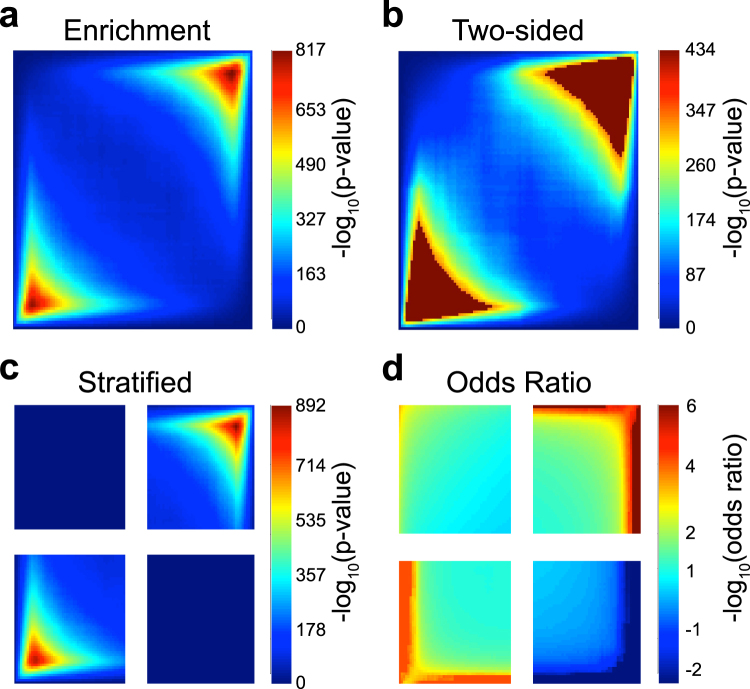
Heatmaps from simulation 1, with both studies showing the same effect size directions. RRHO plots are shown using (**a**) Enrichment, (**b**) Two-Sided, (**c**) Stratified, and (**d)** Log Odds Ratio RRHO methods.

**Figure 5:**
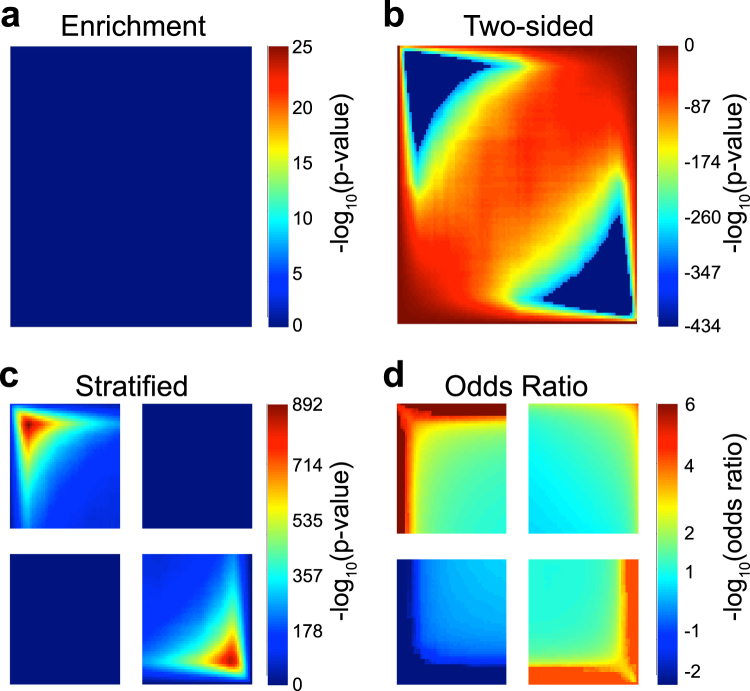
Heatmaps from simulation 2, with the two studies showing opposite effect size directions. RRHO plots are shown using (**a**) Enrichment, (**b**) Two-Sided, (**c**) Stratified, and (**d**) Log Odds Ratio RRHO methods.

We perform the simulation with $S=2\,\,$and σ = 1 (σ = 2, 3, 5, 7 in the supplement) to account for the noise between studies. The R package, *Limma*, is used to compare gene expression levels between control and case groups^8^. The two-sided *p*-values from *Limma* are transformed into one-sided *p*-values that take into account the directions of the estimated effect sizes. The first simulation (Fig. 4) demonstrates effect sizes moving in the same direction (concordant studies). To simulate this we directly calculate the effect size for both studies from a Bernoulli distribution as in step 3b, so the estimated effect size in experiment X has the same direction as the estimated effect size in experiment Y. In simulation 2 (Fig. 5), we demonstrate the case when the effect sizes move in opposite directions. To simulate this, we flip the effect size in only one study. For both simulations, the Stratified representation demonstrates the concordant quadrants having significant overlap between the two experiments.

## Results

### Simulation 1: Same Effect Size Direction

In simulated data 1, the effect sizes were consistent in both studies. That is, the same genes were up-regulated in both studies and the same genes were down-regulated in both studies; simulated data 1 does not contain genes that are up-regulated in one study and down-regulated in the other study. Thus, the RRHO plots using simulated data 1 should only include hotspots in quadrants B (down-regulated in both studies) and C (up-regulated in both studies). The Enrichment method gives an accurate depiction of the overlap in the concordant quadrants. According to Fig. 4a, there is significant overlap in the concordant directions, as expected given the simulated data. While this is an accurate representation, the Enrichment method fails to capture the true overlap in the discordant directions. The upper left and lower right quadrants show no significant overlap (blue heatmap color), but due to the nature of the Enrichment algorithm, the discordant directions cannot be interpreted, so the heatmap color in those quadrants is meaningless. The Two-sided method (Fig. 4b) is also misleading. The concordant quadrants, again, are able to depict the overlap but, as explained in the methods, the overlap heatmap color is meaningless in the discordant quadrants because the overlapping score is positive in all four quadrants. Recall that the goal of the Two-sided method is to demonstrate whether genes are over enriched or under enriched, allowing for a positive or negative overlapping score, respectively. A positive hot spot indicates over enrichment, while a negative hot spot indicates under enrichment. When the scale is positive in the concordant quadrants, as in Fig. 4b, the RRHO heatmap is equivalent to the Enrichment method. Aside from being difficult to interpret, the Two-sided method also has limitations in that the algorithm used in the R package does not include an option for setting the scale to a maximum. Furthermore, the algorithm cannot handle cases where the *p*-value is zero, otherwise the log *p*-value will extend to infinity. In our package, RRHO2, we include an option to fix the scale as well as introduce a truncation to bound negative and positive infinity values by the minimum or maximum log *p*-value. The Stratified method (Fig. 4c), captures the true behavior of the studies without difficulty. We can see that the discordant quadrants do not have significant overlap, while the concordant quadrants show over enrichments. In Fig. 4d, we use effect sizes instead of *p*-values to generate the heatmap; the RRHO map is very similar to the Stratified method in that it is able to accurately portray the lack of overlap among the discordant studies. The scale in Fig. 4d ranges from negative to positive because each pixel now represents the negative log of the odds ratio, not the *p*-value. Using the Odds Ratio method returns a visualization that can be interpreted more conservatively than the *p*-value method. Due to the large number of genes in genomic studies, there is the potential for type-I error. When used in conjunction with the *p*-value method, the Odds Ratio heat map can complement the signal seen in the *p*-value map.

### Simulation 2: Opposite Effect Size Direction

In simulated data 2, the effect sizes are opposite in the two studies. In other words, the same genes that are up-regulated in study 1 are down-regulated in study 2 (and vice versa). Thus, an accurate RRHO plot would have signal in quadrants A (up-regulated study 1, down-regulated study 2) and D (down-regulated study 1, up-regulated study 2). Figure 5 demonstrates the plots generated using each RRHO method. As seen in Fig. 5a, the Enrichment method will give an accurate description of the overlap in the concordant quadrants (no signal), but misleading results in the disconcordant quadrants (shows no signal, although simulated data 2 should have signal). Thus, the Enrichment method does not capture signal when there is overlap in genes changed in opposite directions. The Two-sided method (Fig. 5b) has a similar interpretation for simulated data 2, with no signal in the concordant quadrants (B and C). However, when the discordant quadrants have negative scores and red hot spots, the interpretation of the overlap among genes moving in opposite directions is still inconclusive. In Fig. 5b, however, we do see that the upper left and lower right quadrants are blue. This actually indicates that there is discordant overlap (as confirmed in Fig. 5c), yet the interpretation of the colors is counterintuitive. The Stratified method (Fig. 5c), however, captures the true behavior of the studies. We can see that the discordant quadrants do have significant overlap, while the concordant quadrants do not. The Stratified method can clearly detect the overlap among the discordant studies and becomes particularly useful when biologists are specifically interested in viewing how two studies differ. Figure 5d repeats the same analysis, except using the Odds Ratio method instead of *p*-values to generate the heatmap; the RRHO map is very similar to the Stratified method in that it is able to accurately portray the overlap among the discordant studies. However, as mentioned in simulation 1, the scale in Fig. 5d is much lower than the previous three methods, allowing for a more conservative visualization.

### The Odds Ratio RRHO method is robust

We extended our simulations of both the concordant and discordant datasets to situations with different levels of data noise. As outlined in the methods section, we generated expression values from a multivariate normal distribution. Here, we adjusted the variance parameter, sigma, from the multivariate normal distribution to create more variability within the data. The goal is to simulate real life situations where there is noise that can blur the signal present in the data. For example, microarray and RNA sequencing experiments have a large possibility for random error as well as systematic error that may occur during the actual processes of generating expression values from tissue. Furthermore, outliers are inevitable in most studies, as each organism has a unique genetic profile. In addition to outliers, biological variability is present in most studies. In the real data examples given, the subjects come from different environmental backgrounds and have different ages and sexes. All of these factors will contribute to biological noise and heterogeneity among the samples. A strong statistical model can account for this noise and provide a robust result. Despite the conservative visualization, the Odds Ratio method still provides a robust analysis. In Supplementary Figure 3, we demonstrate that the signal remains robust, even with increasing sigma (i.e., increasing noise). When we increase variability in the data, the Odds Ratio method is able to accurately capture overlap between the two genetic profiles in both the concordance and discordant quadrants.

### Real Data Example – sex differences in human depression

**Figure 6:**
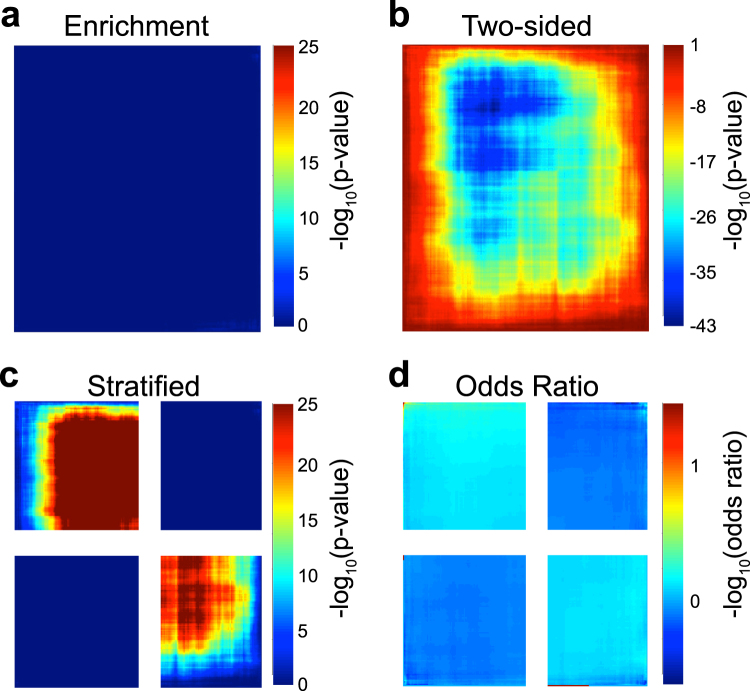
Heatmaps for each RRHO method using real data. (**a**) Enrichment; (**b**) Two-sided; (**c**) Stratified; and (**d**) Log Odds Ratio RRHO methods.

Figure 6 compares results of each RRHO method applied to real data generated by comparing gene expression in the brains of subjects with major depressive disorder (MDD) to control subjects (Brodmann area 11 (BA11); orbitofrontal cortex (OFC)). This data was originally published by Labonte *et al*.^9^ (GSE102556), and the authors used the original RRHO algorithm to assess the overlap in differentially expressed genes between depressed males and females. In Fig. 1c of that manuscript, the RRHO plot indicates no overlap. In a recent publication in which we analyzed this same dataset, we used strict *p*-value cutoffs and identified very few genes that were differentially expressed in both datasets^10^, consistent with Labonte *et al*.^9^. However, we noticed that a majority of the genes that were differentially expressed in both datasets had opposite effect size directions in depressed men and women. Thus, we reasoned that these datasets would provide a valid test for whether the various RRHO algorithms could identify discordant changes across datasets.

The Enrichment method in Fig. 6a shows no red hot spots. This can be interpreted as there being no significant overlap among males and females when the effect sizes of the two studies are concordantly ranked. This result is similar to Fig. 1c in Labonte *et al*.^9^. However, the Enrichment method is unable to detect overlap between the 2 studies in the discordant quadrants. If we only used the Enrichment method, we might conclude that there is no transcriptional overlap in either the concordant or discordant directions between male and female MDD. Using the Two-sided method (Fig. 6b), the plot looks quite different, yet the interpretation of the visualization is unclear. Due to the large presence of red hot spots, one might view Fig. 6b as indicating that there is significant overlap in all four quadrants. However, a red hot spot in the Two-sided method does not indicate significant overlap, but rather the degree of under and over enrichment. The scale of Fig. 6b ranges from −43 to 1; a log_10_(*p*-value) of 1 is not significant, yet the heat map shows a red hotspot. So, when examining the RRHO generated with the Two-sided method, we should instead look for blue spots as indicating transcriptional overlap in the discordant quadrants A and D; this makes the Two-sided plot difficult to interpret. As mentioned in the simulation setting, one limitation with the original RRHO algorithm is that the R package does not provide an option to fix the scale in the Two-sided method. Because of this, the results between the Two-sided and Enrichment methods can seem contradictory.

Our new Stratified method (Fig. 6c) can clearly portray the true overlap between the studies at each effect size direction. Each pixel in the Stratified heatmap still represents a −log_10_(*p*-value) from the hypergeometric distribution. The difference, however, between the Stratified and the Enrichment/Two-sided methods is that each pixel within each quadrant has a clear interpretation with regards to varying effect size directions. The lower left (quadrant C) and upper right (quadrant B) quadrants of Fig. 6c demonstrate a lack of overlap in the concordant directions, which is consistent with the Enrichment result. However, the upper left (quadrant A) and lower right (quadrant D) quadrants reveal that there is significant overlap among the genetic profiles when the effect sizes move in opposite directions. We previously published a modified version of Fig. 6c in the publication in which we describe the findings from our analysis of this same dataset (Supplemental Figure S5 in^10^). Thus, using the Stratified method, we can conclude that there is significant transcriptional overlap in genes changed in opposite directions in male and female depression. This is consistent with our observation that a majority of the genes that are significantly differentially expressed in both males and females have opposite effect size directions^10^. Notably, we would have completely missed this result if we used the previous Enrichment method. Figure 6d visualizes the same data using the Odds Ratio method; recall from the methods section that each pixel on the heatmap now represents the log odds ratio degree of overlap between the two studies. While the color scale is different between the odds ratio and *p*-value methods, the conclusion is the same. We can see that the degree of overlap is more significant in the discordant directions than the concordant directions using the Odds Ratio method (Fig. 6d). Because the scale only ranges from 0 to 1, the Odds Ratio method indicates that while there is discordance across these two datasets, the effect size is not large; this is likely due to the large number of genes in the dataset (~42,000).

One drawback of the methods that utilize the *p*-value visualization (Enrichment, Two-sided, Stratified) is that when the number of genes is large, we can easily get a signal. Furthermore, the scale of the *p*-value method can easily change the interpretation of the heatmap. In Fig. 6c, the scale for the Stratified visualization is fixed at 25. A lower scale would cause these plots to appear more significant, while a higher scale would produce less of a hotspot. Using the Odds Ratio method, the default scale directly corresponds to the degree of overlap and provides a more conservative visualization of the overlap in the discordant directions. Because of this, plotting the odds ratio can provide a completely unbiased visualization of the overlap between the two studies and can be used to compliment the results shown in any *p*-value method. We note, however, that the Odds Ratio method is sensitive to number of genes tested. In other words, the more genes tested, the stronger the signal must be for the Odds Ratio method to detect significant overlap.

We performed a similar analysis with a different published dataset that used the original Enrichment RRHO method and reported significant concordant changes across two datasets^6^. We compared the two DE lists using the RRHO Enrichment method (Supplementary Figure 4a). It is clear that there is concordant overlap among genes, however the discordant directions remain inconclusive (i.e., some apparent signal in discordant quadrants). When using the Stratified method, we can see that the concordant overlap remains as conclusive as when using the Enrichment method, but it is now clear that there is no overlap among the discordant genes (Supplementary Figure 4b). Thus, the Stratified method provides similar results to published studies that reported concordant changes across datasets and a more accurate representation of discordant quadrants.

## Discussion

RRHO offers a promising alternative to previous methods of overlapping Enrichment analysis, such as the one-sided fishers exact test, which uses a single cut-off value. The implementation of RRHO allows researchers to view the overlap enrichment of genes in two biological mechanisms across a gradient of *p*-values. Here, we use both simulations and real experimental data to demonstrate the utility and drawbacks of the existing RRHO methods and how our new “Stratified” method addresses previous drawbacks. A summary of the results across the different RRHO methods is provided in Table 1; for each method, we indicate whether each quadrant is biologically meaningful and/or intuitive to interpret.

**Table 1:** Summary of findings indicating whether each quadrant for the various RRHO methods is biologically meaningful and provides an intuitive representation.

		Enrichment (previous)	Two-sided (previous)	Stratified (current)
Quadrant A (up study 1, down study 2)	Biologically meaningful?	No	Yes	Yes
	Intuitive representation?	No	No	Yes
Quadrant B (down study 1, down study 2)	Biologically meaningful?	Yes	Yes	Yes
	Intuitive representation?	Yes	No	Yes
Quadrant C (up study 1, up study 2)	Biologically meaningful?	Yes	Yes	Yes
	Intuitive representation?	Yes	No	Yes
Quadrant D (down study 1, up study 2)	Biologically meaningful?	No	Yes	Yes
	Intuitive representation?	No	No	Yes

Many recent publications have successfully used RRHO to identify patterns of concordant transcriptional changes in two distinct datasets, and many of these manuscripts are in high profile journals^2,6,9^. When the goal is to identify concordant changes, the original RRHO method was completely valid and our Stratified method yields similar results, as we show in Supplementary Figure 4. In one publication, the authors successfully used their RRHO results to identify large-scale changes in gene expression in response to stress that were shared across brain regions. Since this manuscript used the one-sided test and specifically aimed to identify concordant changes across datasets, the results in that paper are accurate (and provide very interesting and meaningful conclusions)^2^. One gene identified through the cross-brain region RRHO was significantly changed in only one brain region, but manipulation of this gene in the other brain region still induced a change in behavior. A strict differential expression cut-off would have missed the relevance of this gene to mood. The RRHO approach revealed this gene’s importance. Further, this manuscript used the set of overlapping genes across datasets that were identified through RRHO to zero-in on relevant gene ontology (GO) terms. Together, the previous studies that used RRHO with the goal of identifying concurrent changes across datasets provide accurate results on congruency.

Despite the advantages in identifying concordant changes across studies, the original method is limited in its output interpretation when interest lies in how two studies might be discordant. Because of this, many papers using the previous RRHO method provide inconclusive interpretations of their RRHO analyses. Indeed, there are circumstances in which the same genes might be oppositely impacted (i.e., discordant). For instance, one might want to identify novel sets of genes that are up-regulated in a disease state, but down-regulated after successful treatment. Once these overlapping genes are identified using RRHO, follow-up studies using GO pathway analysis can identify biological processes represented by these gene sets. Numerous previous studies have successfully used GO pathways for improving biomarker discovery (e.g.^11–13^).

Our new representation, RRHO2, includes a third “Stratified” option for RRHO analysis. The Stratified method, as demonstrated in the results section using both simulated and real data, offers an intuitive understanding of the strength of enrichment between two biologic processes. The Stratified method heatmap clearly shows 4 quadrants, which can easily be understood as the enrichment overlap between two datasets among all combinations of up- and down-regulated genes. Of note, the experimental dataset we use to show the utility of our Stratified approach was derived from a publication that used the original RRHO algorithm to compare gene expression changes in depressed men and women and reported a lack of both concordant and discordant changes^9^. Their results are similar to what we report with the Enrichment algorithm in Fig. 6a. While the authors were correct to conclude that there was a lack of concordance across the two studies, the original Enrichment RRHO algorithm is unable to inform discordance. When we applied the new Stratified RRHO method, we found a pattern of discordance across the two studies (Fig. 6c). In other words, the same genes were changed in opposite directions in depressed men and women; this finding has important implications for primary pathology and novel treatment development^10^. It was only using the Stratified RRHO method that the discordance between the two studies was identified. We would argue that this has large implications for interpretation of any published studies using the original RRHO algorithm.

The methods section clearly outlines the interpretation of each quadrant using our new RRHO2 Stratified method. We also demonstrate the use of the effect size from the fisher’s exact test to rank the genes. Creating a heatmap using the Odds Ratio method overcomes the obstacles of having a very large sample size, where there is potential for type 1 error.

We provide an R package that is available in Bioconductor (RRHO2) which includes options for all of the analysis methods: Stratified, Two-sided, Enrichment, and the Odds Ratio ranking method. RRHO, when used correctly, provides insight into the degree of overlap between two ranked gene lists. Building on the foundations of RRHO, RRHO2 will further prove that utilizing a gradient to visualize the overlap significance between two genetic profiles allows for remarkable contributions to the fields of genomics and biology.

## Electronic supplementary material

Supplementary Information
