# Supplementary material for: Improved identification of concordant and discordant gene expression signatures using an updated rank-rank hypergeometric overlap approach
Source: Sci Rep. 2018 Jun 25;8:9588. doi: 10.1038/s41598-018-27903-2 (PMC6018631; doi:10.1038/s41598-018-27903-2)
Supplement: Supplementary file 1 — Supplementary Information [file 41598_2018_27903_MOESM1_ESM.pdf]

-- Supplementary Information --

Improved identification of concordant and discordant gene expression signatures using an updated rank-rank hypergeometric overlap approach

Kelly Cahill[1,#], Zhiguang Huo[1,2,#], George Tseng[1,3],
Ryan W. Logan[4,5,6,*], Marianne L. Seney[4,5,*]

[1]Department of Biostatistics, Graduate School of Public Health, University of Pittsburgh, Pittsburgh, PA, USA

[2]Department of Biostatistics, College of Public Health & Health Professions College of Medicine, University of, Gainsville, FL, USA

[3]Department of Computational and Systems Biology, School of Medicine, University of Pittsburgh, Pittsburgh, PA, USA

[4]Department of Psychiatry, School of Medicine, University of Pittsburgh, Pittsburgh, PA, USA

[5]Translational Neuroscience Program, School of Medicine, University of Pittsburgh, Pittsburgh, PA, USA

[6]The Center For Systems Neurogenetics of Addiction, The Jackson Laboratory, Bar Harbor, ME, USA

*Corresponding Authors
#These authors contributed equally to this work.

Supporting Information

Two-sided method

To overcome the limitations of the enrichment method, the original RRHO paper proposed a "two-sided" method. Under the two-sided method, the overlapping *p*-value will be calculated based on the relationship between the observed number of overlap, k, and the expected number of overlap, $\bar{k}$. A two-sided hypergeometric p value is calculated.

$$H(k; s, M, N) = \begin{cases} \sum_{j=0}^{k} h(j; s, M, N) \; k \leq \bar{k} \\ \sum_{j=k}^{s} h(j; s, M, N) \; k > \bar{k} \end{cases}$$

Where $k \leq \bar{k}$ represents under enrichment and $k > \bar{k}$ represents over enrichment. To further distinguish between them, the original RRHO paper further introduce a sign to distinguish under/over enrichment:

$$R(k; s, M, N) = \begin{cases} -|\log_{10} H(k; s, M, N)| \; if \; k \leq \bar{k} \\ +|\log_{10} H(k; s, M, N)| \; if \; k > \bar{k} \end{cases}$$

This sign convention for under/over enrichment involves a new symmetry property as $R(k; s, M, N) = -R(s - k; s, N - M, N)$, which makes the interpretation of quadrant A and D of **Figure 2** potentially interesting. The interpretation for the two-sided method is shown in **Supplementary Figures 1 and 2**. For each red dot, if $k > \bar{k}$, we have the exact same interpretation as the enrichment method (i.e., quadrants B and C are biologically meaningful, but quadrants A and D are not) (**Supplementary Figure 1**). When $k \leq \bar{k}$, however, the overlapping score is negative, so we will have biologically meaningful interpretation in quadrants A and D because of the symmetry property

induced by the sign convention, but not quadrants B and C. Under this scenario, a significant negative overlapping score in quadrant A of **Supplementary Figure 2** represents a significant concurrence of up-regulated genes in study 1 and down-regulated genes in study 2**;** and a significant negative overlapping score in quadrant D of **Supplementary Figure 2** represents a significant concurrence of up-regulated genes in study 2 and down-regulated genes in study 1. However, the interpretation of the heatmap colors can be misleading. A positive overlapping score in a discordant region reveals a red hotspot, however the overlapping areas are actually uninteresting (**Supplementary Figure S1**). Thus, interpretation of results from the two-sided method are challenging, with different rules applying to the concordant and discordant quadrants.

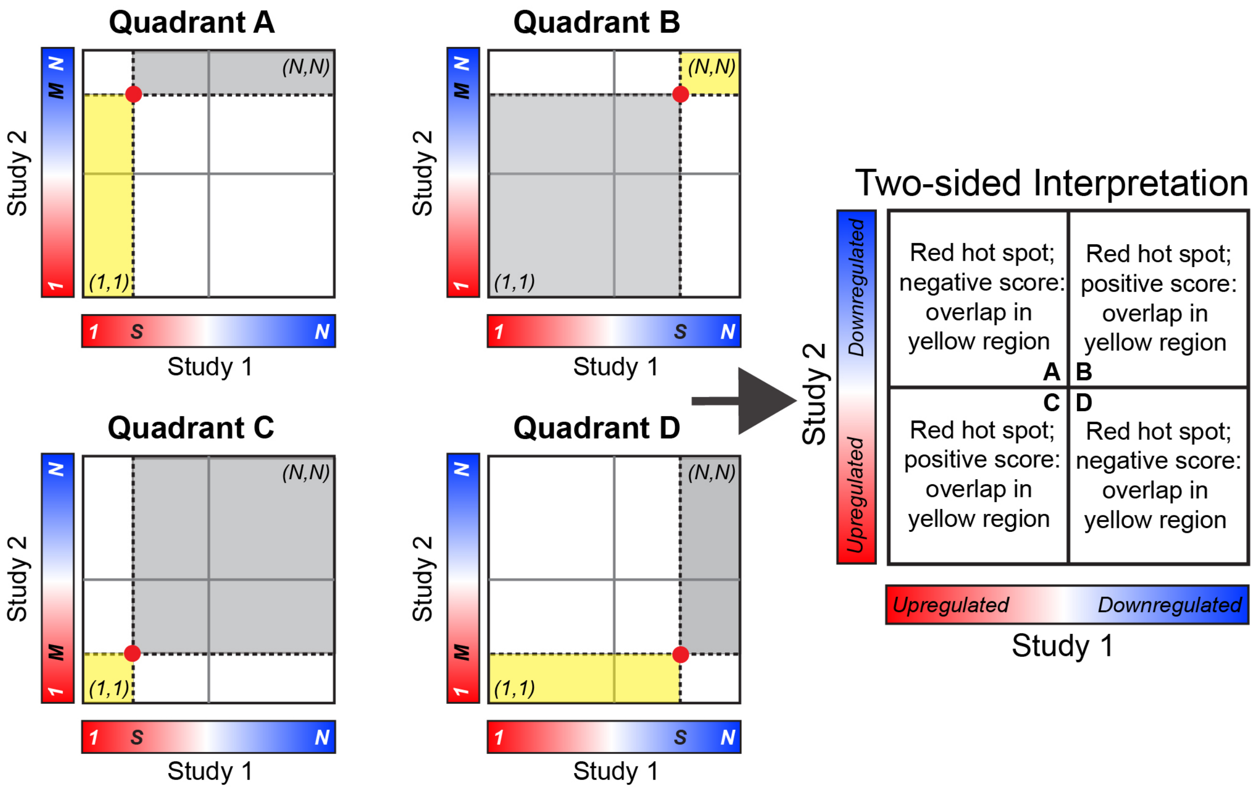

Supplementary Figure 1. Interpretation of the two-sided RRHO method when the *p*-value score is positive. When $k > \bar{k}$, we have the exact same interpretation as the enrichment method (i.e., quadrants B and C are biologically meaningful, but quadrants

A and D are not). When $k \leq \bar{k}$, the overlapping score is negative, so we will have biologically meaningful interpretation in quadrants A and D, but not quadrants B and C. Additionally, a positive overlapping score in quadrants A and D might show a red hotspot, but the overlapping areas are actually not interesting.

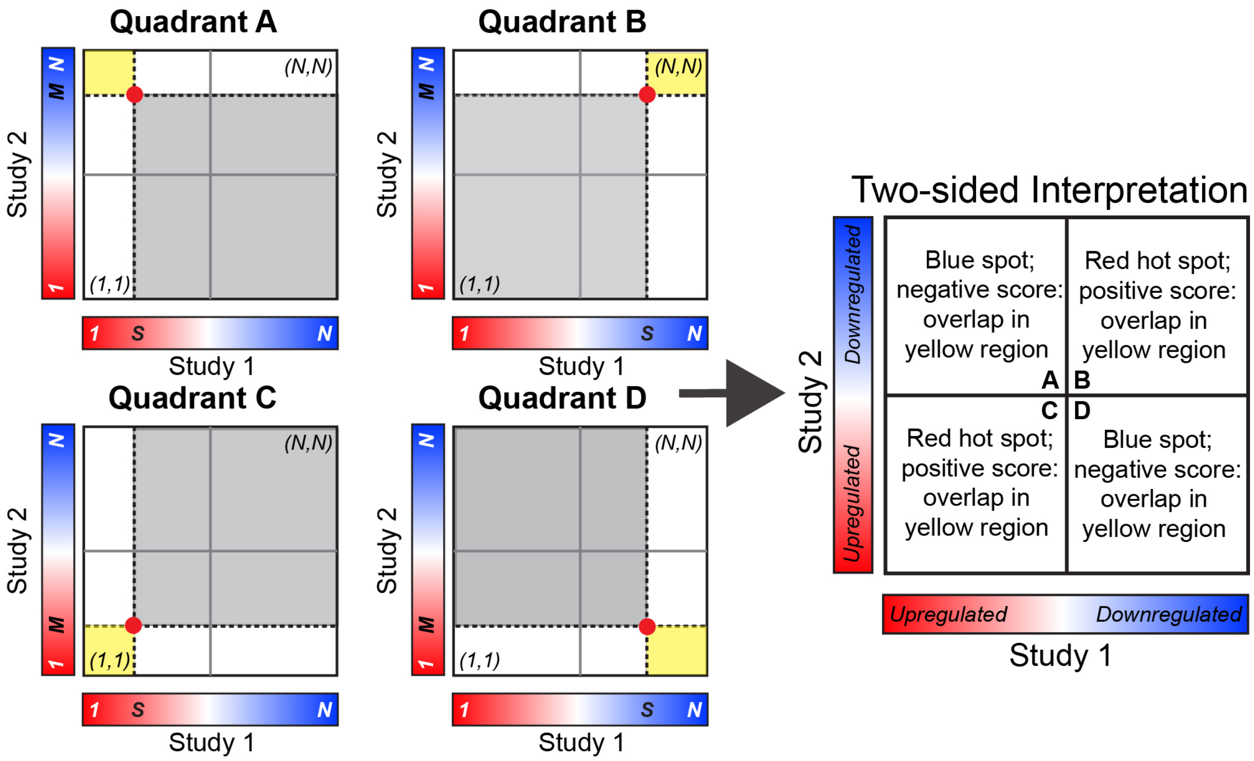

Supplementary Figure 2. Interpretation of discordant quadrants in two-sided RRHO method. For discordant quadrants A and D, a negative *p*-value score in addition to a blue hotspot indicates overlap of genes in the yellow areas. This is different from concordant quadrants B and C, which need positive *p*-values scores and red hotspots to indicate significant overlap. Thus, the two-sided method does indeed provide accurate information for the discordant quadrants; however, the interpretation is difficult.

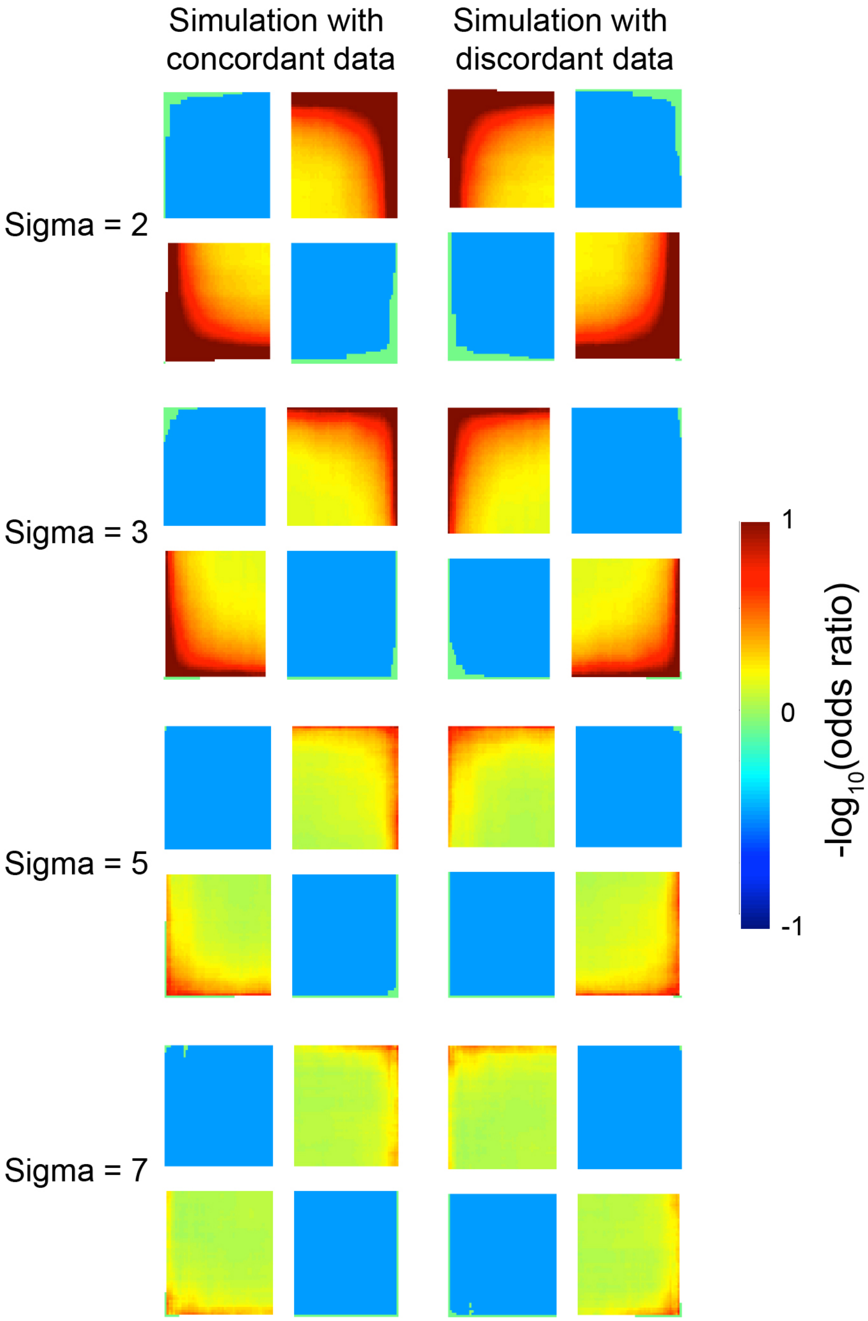

Supplementary Figure 3. Concordant and discordant simulation using the effect size RRHO method. We show that this method provides robust signal, even as the sigma (noise) value increases.

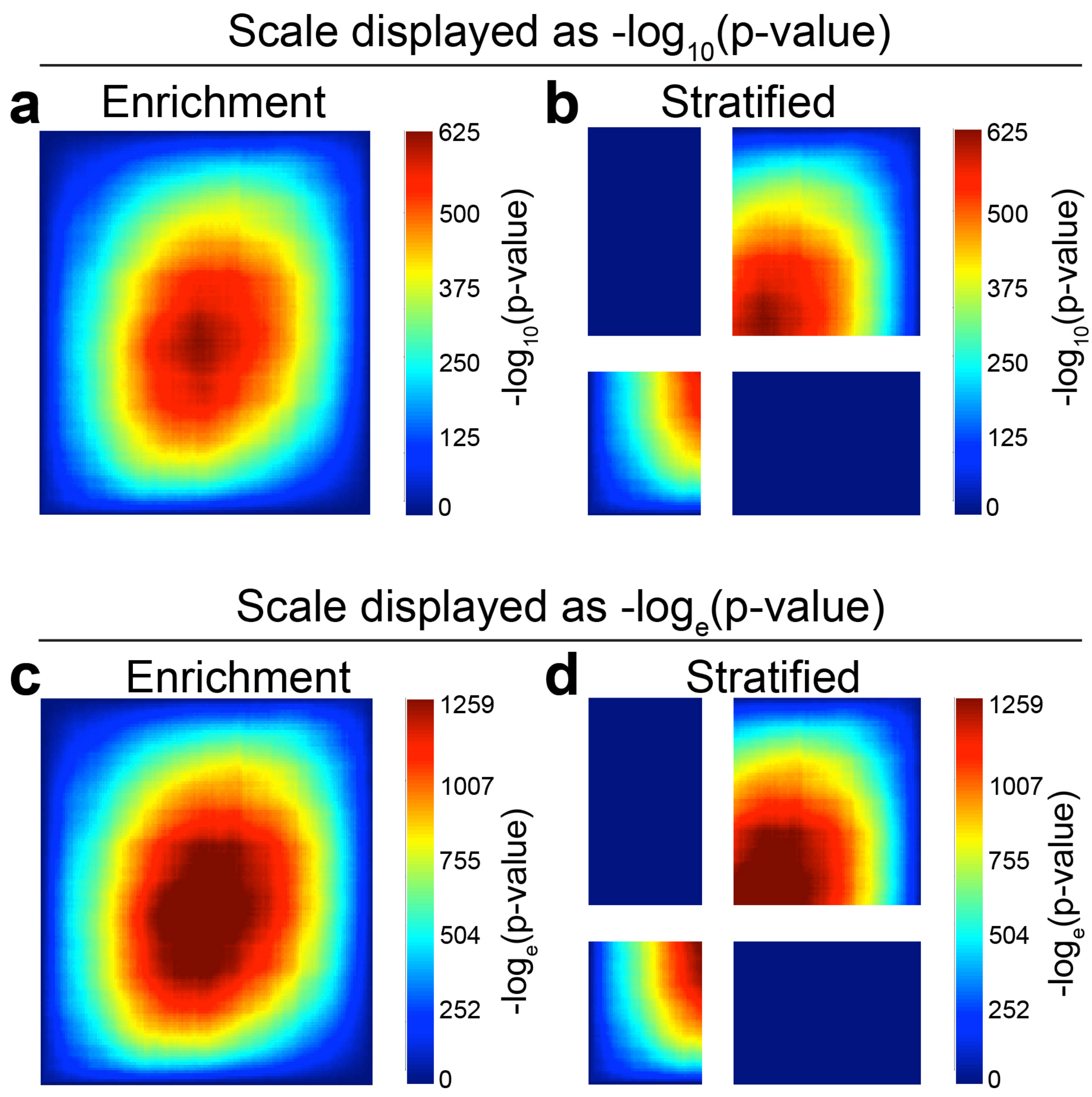

Supplementary Figure 4. Heatmaps generated using real data[1] (a) Enrichment using $-\log_{10}$(*p*-value) scale; (b) Stratified method using $-\log_{10}$(*p*-value) scale; (c) Enrichment using $-\log_{e}$(*p*-value) scale; (d) Stratified method using $-\log_{e}$(*p*-value) scale.

References

1 Pena, C. J. *et al.* Early life stress confers lifelong stress susceptibility in mice via ventral tegmental area OTX2. *Science* **356**, 1185-1188, doi:10.1126/science.aan4491 (2017).
